# The effects of mandibular advancement appliance therapy on the sequence of jaw-closing muscle activity and respiratory events in individuals with obstructive sleep apnea

**DOI:** 10.1007/s11325-022-02624-z

**Published:** 2022-04-28

**Authors:** Deshui Li, Ghizlane Aarab, Frank Lobbezoo, Patrick Arcache, Gilles J. Lavigne, Nelly Huynh

**Affiliations:** 1 Department of Orofacial Pain and Dysfunction, Academic Centre for Dentistry Amsterdam (ACTA), University of Amsterdam and Vrije Universiteit Amsterdam, Amsterdam, The Netherlands; 2 Faculté de Médicine Dentaire, Université de Montréal, Montreal, Canada

**Keywords:** Obstructive sleep apnea, Jaw-closing muscle activity, Respiratory event, Sequence, Mandibular advancement appliance

## Abstract

**Purpose:**

To determine the effects of a mandibular advancement appliance (MAA) on sequences of jaw-closing muscle activity (JCMA) and apneic or hypopneic event (AHE) in individuals with obstructive sleep apnea (OSA).

**Methods:**

Individuals with OSA were included in a secondary analysis of a randomized controlled crossover trial, in which two ambulatory polysomnographic recordings were performed: one with MAA in situ and the other without MAA. A time span of 16 s between JCMA and AHE was applied to classify JCMAs into four sequences: (1) JCMA occurs before AHE (B-type); (2) both events occur simultaneously (S-type); (3) JCMA occurs after AHE (A-type); and (4) JCMA is time-unrelated to AHE (U-type). The effects of MAA on the distribution of these sequences were analyzed by Wilcoxon signed-rank test.

**Results:**

Among 16 individuals (10 men, mean age 51.3 ± 8.5 years) baseline apnea–hypopnea index and JCMA index were 23.8 ± 16.0 events/h and 10.8 ± 10.3 events/h, respectively. In both conditions, i.e., without and with MAA, most JCMAs were U-type (48% and 65%, respectively), followed by A-type (41% and 22%), B-type (25% and 21%), and S-type (2% and 1%). With MAA in situ, only the A-type JCMA index decreased significantly (*P* = 0.005), while B-type, S-type, and U-type JCMA indices did not change significantly (all *P* > 0.05).

**Conclusion:**

MAA therapy only significantly reduces the jaw-closing muscle activities that occur after apneic or hypopneic events in individuals with OSA.

**Trial registration:**

www.clinicaltrials.gov (NCT02011425); December 13, 2013.

## Introduction

Obstructive sleep apnea (OSA) is a sleep-related breathing disorder characterized by repetitive obstructions of the upper airway that may result in oxygen desaturations and arousals from sleep [1]. OSA is usually accompanied by loud snoring, morning headache, and excessive daytime sleepiness [1, 2]. The overall population prevalence of OSA ranges from 9 to 38%, and is higher in individuals with male gender, higher age, and obesity [3]. OSA has been reported to be a risk factor for several metabolic (e.g., diabetes, glucose dysregulation), cardiovascular (e.g., hypertension, stroke), psychiatric (e.g., depression), and sleep-related disorders (e.g., sleep bruxism, insomnia) [4, 5].

Jaw-closing muscle activity (JCMA) is an increased electromyography activity that commonly occurs during sleep in individuals with OSA [6]. JCMA includes rhythmic masticatory muscle activity (RMMA, i.e., muscle activity characterizing sleep bruxism) and orofacial activity (e.g., swallowing, yawning, lip movement, and sleep talking).

Previous studies suggested that in individuals with OSA, respiratory events (apneic or hypopneic events [AHEs]) are frequently followed by JCMA [7, 8], and JCMAs’ onset may be triggered by the AHEs [9, 10]. Two studies reported that the majority of RMMAs occurred after AHEs in individuals with OSA [11, 12], supporting a theory that JCMA in association with the termination of AHE may play a protective role by re-opening the upper airway [13]. However, other studies showed that most RMMAs were time-unrelated to AHEs [14, 15]. Therefore, the temporal relationship between JCMA and AHE in individuals with OSA is probably not characterized by one specific sequence of events at the level of the individual patient [13].

Additionally, some studies suggested that JCMA is a general motor response to sleep arousals [16–18]. In our primary study [19], in which the same sample was used as in the present study, we observed that the effect of a mandibular advancement appliance (MAA) significantly reduces JCMAs related to respiratory arousals in participants with OSA [19]. Based on this, we hypothesized that only JCMAs occurring after AHEs would decrease with MAA treatment, while JCMAs occurring before AHEs, during AHEs, and those time-unrelated to AHEs would not change. Furthermore, we hypothesized that only JCMAs occurring after AHEs in relation to respiratory arousals would decrease. Therefore, the primary aim of this study was to determine the effects of MAA on the distribution of sequences of JCMAs and AHEs. The secondary aim was to determine the effect of MAA on JCMA occurring after AHE in relation to arousal.

## Methods

### Study design

This study is a secondary analysis of a prospective randomized controlled crossover trial in which the effects of MAA therapy on JCMA in individuals with OSA were investigated [19]. This clinical study is registered at www.clinicaltrials.gov (NCT02011425). The scientific and ethical aspects of this study were approved by the Medical Ethics Committee of the Université de Montréal (13–105-CERES-D).

### Participants

Individuals who were prescribed MAA therapy by a physician for their OSA were recruited in the primary study. The criteria of participants’ recruitment were described in detail by Aarab et al. [19]. In summary, participants aged between 30 and 65 years with an apnea–hypopnea index (AHI) of 15–45 events/h of sleep and OSA signs or symptoms (e.g., choking or gasping during sleep, daytime fatigue) were included. The exclusion criteria were as follows: presence of other respiratory or sleep disorders (except sleep bruxism), ongoing periodontal problems, reversible upper airway abnormalities, severe orofacial pain, or temporomandibular disorders; usage of medications that could influence respiratory or sleep; and lack of retention possibilities for an MAA.

### Polysomnography

After a 3 to 6 months’ habituation period of wearing MAA (SomnoDent Flex; SomnoMed, Ontario, Canada), polysomnographic (PSG) recordings were conducted for participants at two conditions, i.e., without and with MAA in situ, in random order with an interval of 1 week to eliminate possible carryover effects. An ambulatory type II PSG system, Embla Titanium hardware (Embla, Ontario, Canada), was used to record the following channels: electroencephalography (F3M2, F4M1, C3M2, C4M1, O1M2, O2M1), electrooculography (left and right), electromyography (EMG; mentalis, masseter, temporalis, and tibialis muscles), airflow (nasal cannula), respiratory effort (abdominal and thoracic), oximetry, and sleep position.

PSG recordings, including standard sleep variables, respiratory events (e.g., apnea, hypopnea), and sleep arousals, were scored manually by an experienced and registered polysomnographic technologist from an independent company (Sleep Strategies, Ottawa, Canada), following the criteria of the American Academy of Sleep Medicine [20]. JCMAs (i.e., RMMA and orofacial activity) were scored by the first author (D.L.) according to previously published criteria [21]. EMG burst was scored when the mean amplitude was two times higher than the baseline EMG signal on at least three of the four EMG channels of the bilateral masseter and temporalis muscles. EMG burst was classified as phasic (duration: 0.25–2 s) or tonic (duration ≥ 2 s). EMG bursts occurring with an interval of shorter than 3 s were considered belonging to a single episode. Subsequently, an RMMA episode was scored as phasic (three or more continuous phasic EMG bursts), tonic (one or more tonic EMG bursts), or mixed (at least one phasic and one tonic EMG bursts). Orofacial activity was scored when EMG bursts did not meet the criteria for RMMA. The number of JCMA was defined as the sum of RMMA and orofacial activity.

### The sequence of JCMA and AHE

**Fig. 1:**
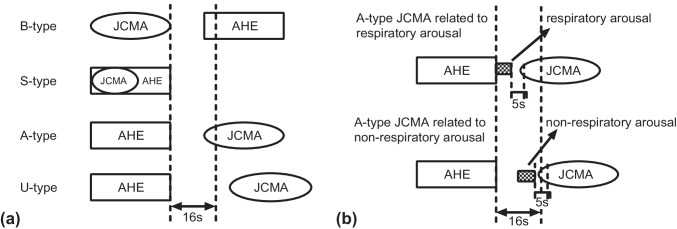
Relationship between jaw-closing muscle activity (JCMA), arousal, and apneic or hypopneic event (AHE). **a** Sequences of JCMA and AHE; **b** Respiratory or non-respiratory arousal-related JCMA. B-type, JCMA occurs before AHE; S-type, JCMA and AHE occur simultaneously; A-type, JCMA occurs after AHE; U-type, JCMA occurs before or after AHE but is time-unrelated to AHE

Scored JCMAs were categorized into four possible sequences in association with AHE (see Fig. 1a): (1) JCMA occurs before AHE (B-type); (2) JCMA and AHE occur simultaneously (S-type); (3) JCMA occurs after AHE (A-type); and (4) JCMA is time-unrelated to AHE (U-type). A time span of 16 s [16, 22–24] was applied for the relation between the two events, starting at the termination of the preceding AHE or JCMA. When JCMA occurred between two AHEs and both time spans were within 16 s, the JCMA was scored twice, i.e., B-type and A-type. Consequently, the total percentages of the four types may be over 100%.

A-type JCMAs were considered in relation to arousal when they occurred within 5 s of the arousal [16, 19]. If the arousal occurred at the termination of or immediately after a respiratory event (i.e., AHE), the arousal was scored as respiratory arousal [25], and the JCMA was then scored as respiratory arousal-related JCMA [16, 19]^.^ In contrast, if the arousal was scored as non-respiratory arousal, the JCMA was scored as non-respiratory arousal-related JCMA (see Fig. 1b).

### Outcome variables

Some variables were transformed into indices, defined as the number of events per hour of sleep, such as the JCMA index. The primary outcome variables of this study were the indices of each sequence of JCMA, viz., B-type JCMA index, S-type JCMA index, A-type JCMA index, and U-type JCMA index. In order to compare our results with other studies, the number of JCMAs for each sequence was also expressed as a percentage of the total number of JCMAs. Secondary outcome variables were the index of A-type JCMA in relation to respiratory and non-respiratory arousal.

### Statistical analysis

The normality of variables was tested by the Shapiro–Wilk test. Paired-samples *t*-tests or Wilcoxon signed-rank tests were used to compare variables between PSG recordings without and with MAA in situ. The effects of MAA on the indices of the four sequences of JCMA and the indices of A-type JCMA in relation to arousals were analyzed by the Wilcoxon signed-rank test. Statistical significance was set at 0.05. Statistical analysis was performed using SPSS Statistics (version 26, SPSS Inc., Chicago, IL, USA).

## Results

### Participants

Thirty-two individuals with OSA were initially invited to participate. For various reasons, fourteen individuals were excluded in the primary study of Aarab et al. [19]. In two participants, the scored AHEs were invisible on the respiratory traces of their PSG recordings. Hence, in this study, we included 16 participants, including 10 men. Mean age was 51.3 ± 8.5 years. Mean body mass index was 29.1 ± 3.6 kg/m^2^. Although all participants met the AHI criteria (i.e., 15–45 events/h) during their recruitment, 6 cases showed an AHI below 15 events/h in the PSG recordings without MAA in situ.

### Descriptive analyses

Table 1 shows the descriptive analyses of the sleep, respiratory, and JCMA variables in PSG recordings without and with MAA in situ. The total sleep time and sleep efficiency did not show a significant difference between PSG recordings without and with MAA in situ. The percentage of sleep stage N2 decreased significantly with MAA in situ, while the percentage of stage N3 and REM increased significantly (*P* < 0.05). The AHI, oxygen desaturation index, total arousal index, and respiratory arousal index decreased significantly with MAA in situ (*P* < 0.05).

**Table 1:** Polysomnographic variables of 16 patients with OSA without and with MAA in situ

	Without MAA^a^	With MAA^a^	Statistics	*P* value
**Sleep variables**				
Total sleep time (min)	359.6|392.8|434.5	357.8|391.5|447.7	*Z* = − 0.259	0.796^b^
Sleep efficiency (%)	88.5 ± 7.0	87.0 ± 6.0	*T* = 0.643	0.530^c^
N1 (%)	87.0 ± 6.0	10.2 ± 3.8	*T* = 1.565	0.138^c^
N2 (%)	66.2 ± 4.5	61.8 ± 5.9	*T* = 2.710	0.016^c^,*
N3 (%)	0.3|2.7|6.8	2.6|7.2|9.4	*Z* = − 2.166	0.030^b^,*
REM (%)	18.7 ± 4.0	21.5 ± 5.5	*T* = − 2.210	0.043^c^,*
Total arousal	14.2 ± 5.6	18.7 ± 7.5	*T* = 2.726	0.016^c^,*
Respiratory arousal	4.0|7.7|14.5	2.5|3.8|8.0	*Z* = − 2.844	0.004^b^,*
Non-respiratory arousal	6.2|8.2|9.3	6.6|8.0|10.5	*Z* = − 0.233	0.816^b^
**Respiratory variables (events/h)**				
AHI	14.3|19.8|31.7	6.4|13.9|23.9	*Z* = − 2.947	0.003^b^,*
Oxygen desaturation index	14.2|22.8|28.8	6.8|14.9|26.6	*Z* = − 2.443	0.015^b^,*
**JCMA variables (events/h)**				
Total JCMA	4.1|6.7|9.4	2.5|3.9|6.4	*Z* = 1.215	0.234^b^
RMMA	0.8|2.0|3.6	0.6|1.0|2.0	*Z* = 1.034	0.277^b^
Orofacial activity	2.3|4.5|5.8	1.5|2.7|4.6	*Z* = 1.212	0.061^b^

^a^For normally distributed variables, data are presented as mean ± one standard deviation; for non-normally distributed variables, the 25%|50% (median)|75% percentiles are given

^b^Wilcoxon signed-rank test

^c^Paired samples *t*-test

^*^Statistically significant at the 0.05 probability level

*OSA*, obstructive sleep apnea; *MAA*, mandibular advancement appliance; *AHI*, apnea–hypopnea index; *RMMA*, rhythmic masticatory muscle activity; *JCMA*, jaw-closing muscle activity (JCMA = RMMA + orofacial activity)

### Sequences of JCMA and AHE

Table 2 shows the distribution of each sequence of JCMA and AHE without and with MAA in situ. In both conditions, i.e., without and with MAA in situ, the majority of JCMAs were classified as U-type (mean = 48% and 65%, respectively), followed by A-type (mean = 41% and 22%), and B-type (mean = 25% and 21%). Only a few JCMAs were scored as S-type (mean = 2% and 1%). In addition, without MAA, 16% (mean) of JCMAs were scored as both A-type and B-type, while with MAA in situ, the percentage of the double-scored JCMAs decreased to 8%.

**Table 2:** Distribution of sequences of jaw-closing muscle activity and apneic or hypopneic events in patients with OSA

Sequences	Without MAA		With MAA		*Z*^c^	*P* value
	Index^a^ (events/h)	Percentage^b^ (%)	Index^a^ (events/h)	Percentage^b^ (%)		
B-type	0.2|1.4|3.9	25 ± 22	0.3|0.8|1.5	21 ± 17	− 1.817	0.069
S-type	0.0|0.0|0.2	2 ± 3	0.0|0.0|0.2	1 ± 2	− 0.840	0.401
A-type	0.5|2.7|5.6	41 ± 25	0.2|1.2|1.8	22 ± 15	− 2.783	0.005*
U-type	1.2|3.1|4.0	48 ± 28	1.5|2.2|5.7	65 ± 22	− 0.672	0.501

^a^Data are presented as percentiles (25%|50% (median)|75%)

^b^Data are presented as mean ± one standard deviation

^c^Statistical analysis was performed by the Wilcoxon signed-rank test based on the indices of each sequence

^*^Statistically significant at the 0.05 probability level

*MAA*, mandibular advancement appliance; *JCMA*, jaw-closing muscle activity; *B-type*, JCMA occurs before apneic or hypopneic event (AHE); *S-type*, JCMA and AHE occur simultaneously; *A-type*, JCMA occurs after AHE; *U-type*, JCMA is time-unrelated to AHE

**Fig. 2:**
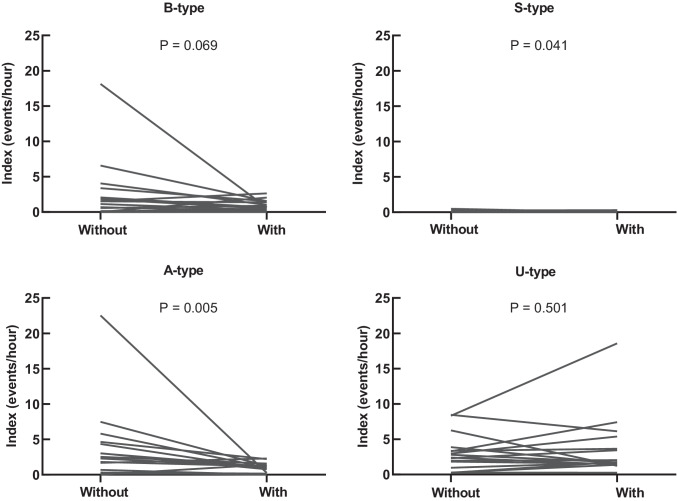
Individual values of JCMA index for each sequence without and with MAA in individuals with OSA. *A *P* value of < 0.05 is considered statistically significant; MAA, mandibular advancement appliance; JCMA, jaw-closing muscle activity; B-type, JCMA occurs before AHE; S-type, JCMA and AHE occur simultaneously; A-type, JCMA occurs after AHE; U-type, JCMA occurs before or after AHE but is time-unrelated to AHE

With MAA in situ, only the A-type JCMA index decreased significantly compared with that without MAA (*P* = 0.005), while B-type, S-type, and U-type JCMA showed no significant difference (*P* = 0.069, 0.401, and 0.501, respectively, see Table 2). This finding still holds after removing the double-scored JCMAs from A-type and B-type (for A-type, *P* = 0.023; for B-type, *P* = 0.326). Although the reduction of the B-type JCMA index was not significant, 10 of the 16 participants showed a decrease (Fig. 2). Also, in a few cases, the A-type, B-type, and U-type JCMA index increased with MAA in situ.

In addition, for the A-type JCMA index, only the respiratory arousal-related JCMA index decreased significantly with MAA in situ (0.40|2.15|3.67 vs 0.16|0.57|0.75, *P* = 0.001), whereas the non-respiratory arousal-related JCMA index did not show a significant difference (0.00|0.21|0.71 vs 0.00|0.06|0.37, *P* = 0.170).

## Discussion

This study aimed to determine the effects of MAA on the distribution of the sequences of JCMA and AHE in individuals with OSA. Our results showed that MAA therapy significantly reduced only the JCMAs that occurred after AHEs in relation to respiratory arousals; not those that occurred after AHEs in relation to non-respiratory arousals, nor those that occurred before AHEs, during AHEs, or were time unrelated to AHEs.

A recent study demonstrated that both RMMA and other orofacial activities are involved in a cascade of arousal-related motor responses during sleep [26]. Considering that sleep arousals commonly follow AHEs in OSA, it can be assumed that RMMA and other orofacial activities have similar temporal relationship to AHEs. Also, the reliable distinction between RMMA and orofacial activities relies on audio–video recordings [21]. However, the PSG used in this study did not include such recordings. In addition, as previous studies on the temporal relationship between RMMAs/orofacial activities and AHEs are rare, especially for orofacial activity, comparisons between our study and others would be limited. For these reasons, in this study, we combined both types of oromotor events as JCMA to avoid potential bias as well as to analyze their temporal relationship to AHEs.

Currently, there is no evidence pointing out an appropriate time span to consider JCMA and AHE as being related [13]. Based on evidence gathered from several sources, we set the time span at 16 s. Hosoya et al. performed a PSG study to investigate the relationship between sleep bruxism and OSA, which concluded that RMMA is secondary to arousal that occurs after AHE [22]. Based on this, the time span between AHE and RMMA was regarded as involving three periods: (1) the time span between AHE and arousal; (2) the duration of arousal; and (3) the time span between arousal and RMMA. Based on the results of three studies, the time of the three periods was determined at 0.9 s [23], 10.8 s [24], and 4 s [16], respectively. Thus, by adding up the times of the three periods, the time span of AHE to RMMA was estimated to be 16 s. Based on this, when RMMA occurred after AHE within 16 s, we took these two events as being related and classified this RMMA into A-type. Unfortunately, to the best of our knowledge, available evidence regarding the possible time span between AHE and JCMA seems to be only available for RMMA that occur after AHE; not for RMMA that occur before AHE. Similarly, such evidence seems to be unavailable for orofacial activities. Therefore, in this study, we applied the 16-s rule to all types of JCMA and all possible sequences.

Theoretically, a longer time span would result in more JCMAs classified as AHE-related, i.e., B-type and A-type. This is indeed the case when comparing our results with those from Tsujisaka et al. [15] and those from Saito et al. [11]. In the study of Tsujisaka et al. [15], the 10-s rule resulted in around 40% of RMMAs and 18% of orofacial activities being related to AHEs, while the 16-s rule in our study resulted in 52% of JCMAs and the 5-min rule in the study of Saito et al. [11] even resulted in 80% of RMMA being related to AHEs.

Another possible reason for the discrepancy between these studies may be the participants’ OSA severity. The present study was composed of participants with OSA ranging from moderate to severe, while in the study of Tsujisaka et al. [15], which had a time-span setting comparable to ours, only mild cases were included. Also, our study showed more JCMAs that were associated with AHEs than the study of Tsujisaka et al. [15]. Based on this, we speculate that in severe cases, more JCMAs would be related to AHEs than in mild cases. This notion could support, at least partially, the expert opinion that the relative predominance of one specific sequence of events varies at the level of the individual patient [13]. Future studies are needed to confirm this hypothesis.

Corresponding to the four possible sequences of JCMA and AHE, four theories of the role of JCMA in OSA were hypothesized [13], viz., (1) B-type: JCMA may have an OSA-inducing effect; (2) S-type: the genesis of two events may share the same stimulus and mechanism; (3) A-type: JCMA may have a potential OSA-protective role; and (4) U-type: two events are unrelated. Furthermore, a possible predominant sequence would support one of these four theories. According to our results (Table 2), the most common JCMA was U-type (48.3%). On the one hand, the result means that around half of the JCMAs were time-unrelated to AHEs. On the other hand, it indicates that the other half of JCMAs were time-related to AHEs (i.e., B-type, A-type, or S-type). Thus, we could not conclude that these two events are unrelated. Since only a few JCMAs were scored as S-type, the presence of S-type seems like a coincidental occurrence of JCMA and AHE. Besides, 25.1% of JCMAs were scored as B-type. However, considering that part of B-type had an overlap with A-type JCMAs and that 10 of the 16 participants showed a reduction in B-type JCMA index with MAA therapy, these overlapping B-type JCMAs were more likely responses to the preceding AHEs and respiratory arousals. Given this, if we subtract the number of overlapping JCMAs (15.5%), the percentage of remaining B-type JCMA will be around 10%, which weakens the rationality of the hypothesis that JCMA has an OSA-inducing effect. Finally, 40.1% of JCMAs were scored as A-type, and most A-type JCMAs were related to respiratory arousals. These results suggest that A-type JCMA is a response to preceding AHE and respiratory arousal, supporting the hypothesis that some JCMAs may have a positive protective role against OSA [13].

Based on our results, we accepted the hypothesis that only A-type JCMA would decrease with MAA therapy, while B-type, S-type, and U-type JCMA would not change. Also, we accepted our second hypothesis that only JCMA occurring after AHE in relation to respiratory arousal would decrease with MAA treatment. These results imply that with MAA therapy, the reduction of the A-type JCMA index is mainly due to the decrease of respiratory arousal-related A-type JCMA. Besides, our results showed that with MAA in situ, only respiratory arousals decreased significantly; not non-respiratory arousals. Considering all this evidence, we can speculate that successful MAA treatment may effectively reduce JCMA through decreasing respiratory arousal. Furthermore, the efficacy of MAA in reducing JCMA in individuals with OSA may vary at an individual level depending on the proportion of A-type JCMA related to respiratory arousal in the total JCMA.

Additionally, previous studies reported that in some cases, MAA might not be effective in managing OSA, or even aggravate the condition of OSA [26, 27]. Given this, it is not surprising that a few cases in our sample showed an increase in the A-type JCMA index. Furthermore, it could be hypothesized that MAA responders may show a higher reduction in the JCMA index than non-responders in individuals with OSA. Similar to A-type, the U-type JCMA index also displayed an increase in several cases with MAA in situ, suggesting that MAA may increase the frequency of JCMA, even in individuals without OSA. This has also been reported in previous studies [28, 29].

Although this study was performed in participants with OSA and not in a population with comorbid sleep bruxism, based on our results and considering the fact that RMMA is a common muscle activity observed in both OSA and healthy individuals [30], we hypothesize that only RMMAs that occur after AHEs in relation to arousals would be improved by MAA therapy in individuals with OSA. Also, the proportion of respiratory arousal-related A-type RMMAs may be able to predict the efficacy of MAA on reducing the comorbid sleep bruxism in individuals with OSA. Future studies are needed to verify these hypotheses in individuals with both OSA and sleep bruxism.

Although this study provides new findings on the relationship between JCMA and AHE, several limitations should be noted. First, we did not perform an a priori sample size calculation. However, based on the post hoc power analysis, a sample of 11 would be enough to detect the difference in the A-type JCMA index with MAA therapy. Thus, the significant reduction of A-type JCMA with MAA in situ found in this study is considered reliable. Second, the time span setting for scoring sequences was based on limited and indirect evidence. A specifically designed study is needed to define a solid evidence-based time span at which JCMA or RMMA and AHE can be considered being related.

## Conclusion

This study showed that effective mandibular advancement appliance therapy in individuals with obstructive sleep apnea reduces only the jaw-closing muscle activities that occur after respiratory events with arousals; not those that occurred after AHEs in relation to non-respiratory arousals, nor those that occurred before AHEs, during AHEs, or were time-unrelated to AHEs. These results suggest that mandibular advancement appliances can relieve jaw-closing muscle activities that are secondary to OSA, and that the efficacy may vary at the level of individual patients depending on the distribution of jaw-closing muscle activities that occur after respiratory events.

## Data Availability

The datasets generated during and/or analyzed during the current study are available from the corresponding author on reasonable request.
